# Supplementary material for: Janus decellularized membrane with anisotropic cell guidance and anti-adhesion silk-based coatings for spinal dural repair
Source: Nat Commun. 2025 Feb 15;16:1674. doi: 10.1038/s41467-025-56872-0 (PMC11829971; doi:10.1038/s41467-025-56872-0)
Supplement: Supplementary file 1 — Supplementary Information [file 41467_2025_56872_MOESM1_ESM.pdf]

Supplementary Information

Janus decellularized membrane with anisotropic cell guidance and anti-adhesion silk-based coatings for spinal dural repair

Xuewei Bi[1,2,3,4 #], Zhinan Mao[4,5#], Linhao Li[1,2]*, Yilin Zhang[2], Lingbing Yang[2], Sen Hou[2], Juan Guan[5], Yufeng Zheng[4], Xiaoming Li[1,2,3]*, Yubo Fan[1,2,3]*

# Xuewei Bi and Zhinan Mao contributed equally to this work.

[1]Innovation Center for Medical Engineering & Engineering Medicine, Hangzhou International Innovation Institute, Beihang University, Hangzhou, 311115, China

[2]Key Laboratory of Biomechanics and Mechanobiology of Ministry of Education, Beijing Advanced Innovation Center for Biomedical Engineering, School of Biological Science and Medical Engineering, and with the School of Engineering Medicine, Beihang University, Beijing, 100191, China

[3]National Medical Innovation Platform for Industry-Education Integration in Advanced Medical Devices (Interdiscipline of Medicine and Engineering), Key Laboratory of Innovation and Transformation of Advanced Medical Devices of Ministry of Industry and Information Technology, Beihang University, Beijing, 100083, China

[4]School of Materials Science and Engineering, Peking University, Beijing, 100871, China

[5]School of Materials Science & Engineering, Beihang University, Beijing 100191, China

*Corresponding authors. E-mail: x.m.li@hotmail.com (Xiaoming Li); linhaoli@buaa.edu.cn (Linhao Li); yubofan@buaa.edu.cn (Yubo Fan)

This PDF file includes:

Supplementary experimental section

Supplementary figures (Figs. S1 to S9)

Supplementary experimental method

Characterization of SilMA and SF

The molecular weight of SF was characterized by western blot analysis. 10% separation gel and 5% concentration gel were prepared firstly, then the 15 μL samples were added into the well. The voltage was adjusted to 80 V and switched to 110 V when the samples runed into the separation gel and a reddish-brown marker appeared. Subsequently, the gel was removed and washed with deionized water. Finally, the Coomassie Bright Blue solution was added to cover the gel until clear bands of protein are visible.

The wide-angle X-ray diffraction (XRD) patterns of the lyophilized SF (10 mm×10 mm) and SilMA (10 mm×10 mm) were conducted using an XRD (D8 Advance, Bruker, Germany) and a Cu Ka radiation source (1.54 Å). The samples were scanned from 2θ = 10° to 60°.

Magnetic resonance imaging

Eight weeks after the operation, scanning was performed using a philips Ingenia 3.0T MRI instrument to evaluate the status of dura mater repair and epidural scar formation. The parameters used in scanning were listed as follows: Driven Equilibrium Turbo Spin Echo (TSE-DRIVE); repetition time/echo time (TR/TE), 1500/80 ms; Fat suppression techniques, SPectral Attenuated Inversion Recovery (SPAIR); Turbo Factor, 11; slice thickness, 1.6 mm; slice gap, 0.0 mm; number of signal averaged (NSA), 2.

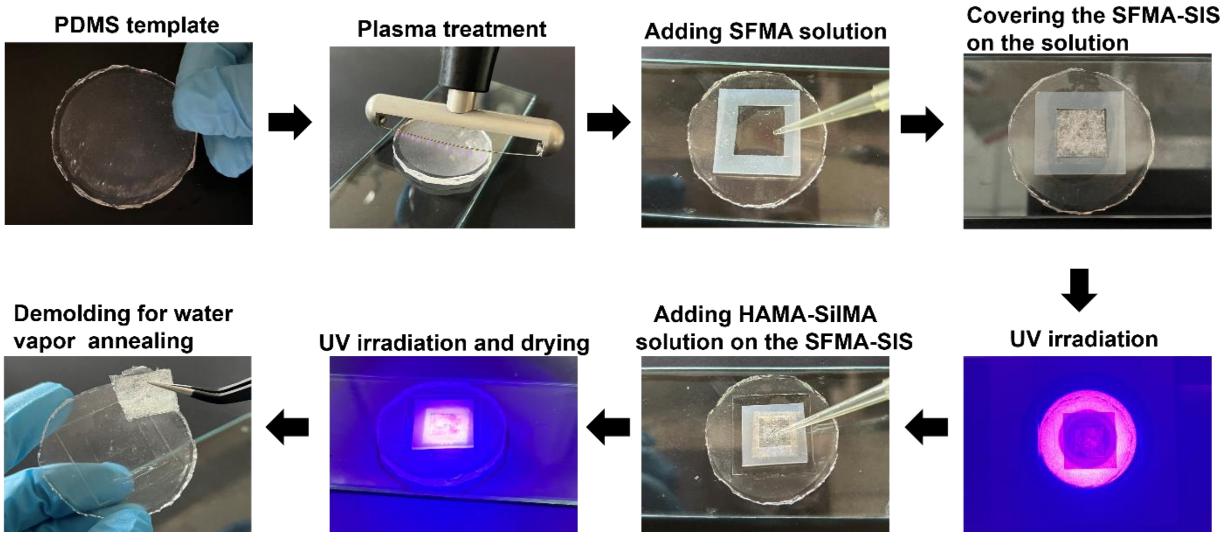

Supplementary Figure 1. Images of the fabrication process of Janus SIS. The PDMS mold with a 20 μm microgroove structure was treated with plasma to endow it with a hydrophilic surface. Then, a silicone frame with an internal diameter of 15 mm x 15 mm was placed on it, followed by the addition of SilMA (60 mg/ml, LAP 0.25%) and SF (60 mg/ml) mix solution (SilMA: SF, 1:1 named SFMA) into the frame. The SFMA-SIS was covered on the solution followed by photocuring with a UV irradiation for 35 s to generate the corresponding microgroove. Next, on the top surface of the SFMA-SIS, SilMA (5 mg/ml, LAP 0.25%) and HAMA (15 mg/ml, LAP 0.25%) mixture was smeared uniformly and irradiated for 15 s and then left to dried at 37 ℃.

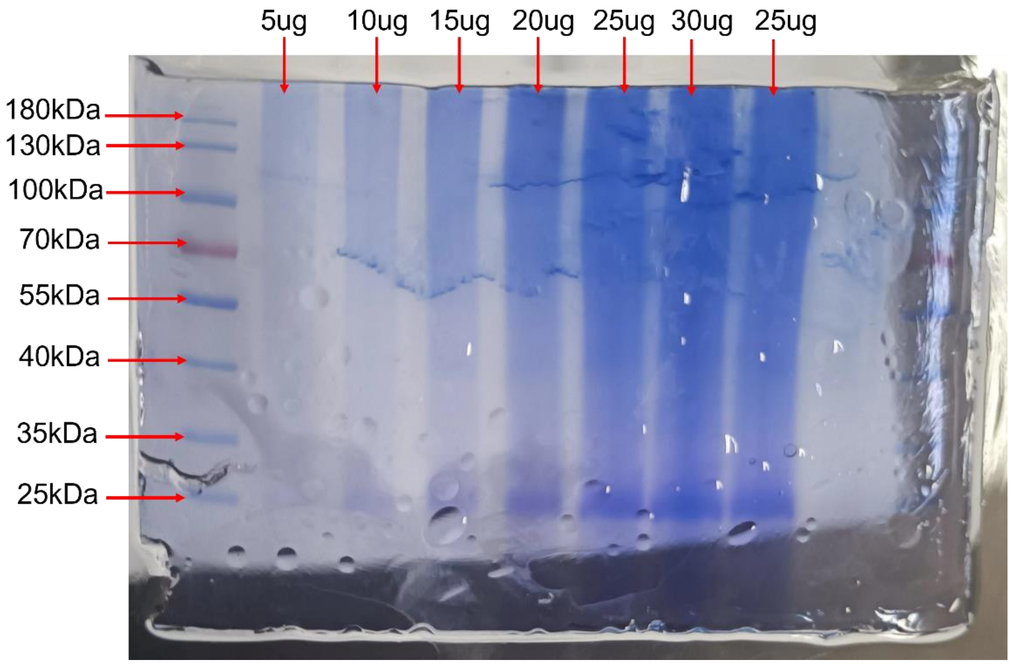

Supplementary Figure 2. Molecular weight distribution of SF measured by western blot.

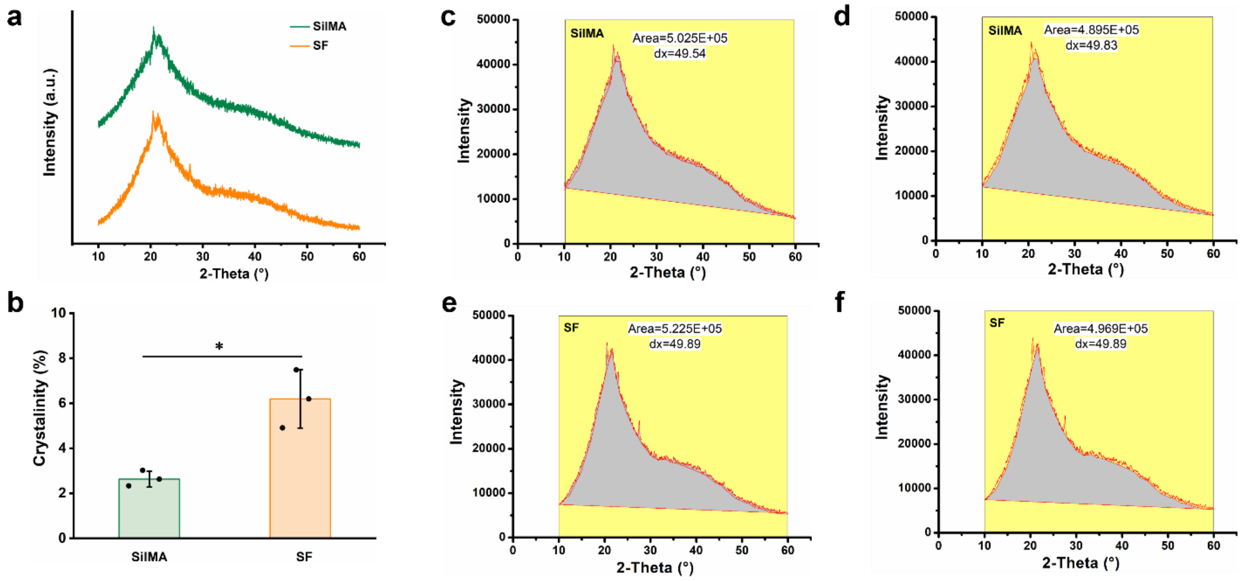

Supplementary Figure 3. Crystallinity Analysis of SilMA and SF. **a** X-ray diffraction patterns of SilMA and SF. **b** Crystallinity of SilMA and SF as calculated by XRD results; Crystallinity (%) = (D - D1) / D × 100%. Values in **b** represent the mean ± SD (*n* = 3 independent samples). **c** The calculation total area D of crystallized and non-crystallized zones of SilMA by using Origin. **d** The calculation area D1 of the non-crystallized zone of SilMA by using Origin. **e** The calculation total area D of crystallized and non-crystallized zones of SF by using Origin. **f** The calculation area D1 of the non-crystallized zone of SF by using Origin. Statistical difference was determined by two-tailed unpaired Student's T-test between two groups. Source data and exact P-values are provided as a source data file. (*$P < 0.05$, **$P < 0.01$, ***$P < 0.001$).

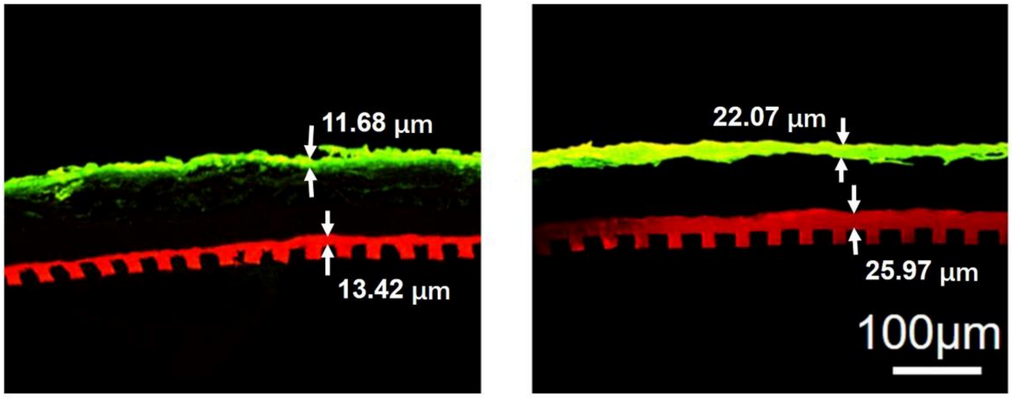

Supplementary Figure 4. CLSM images of Janus SIS membrane with different thickness coating.

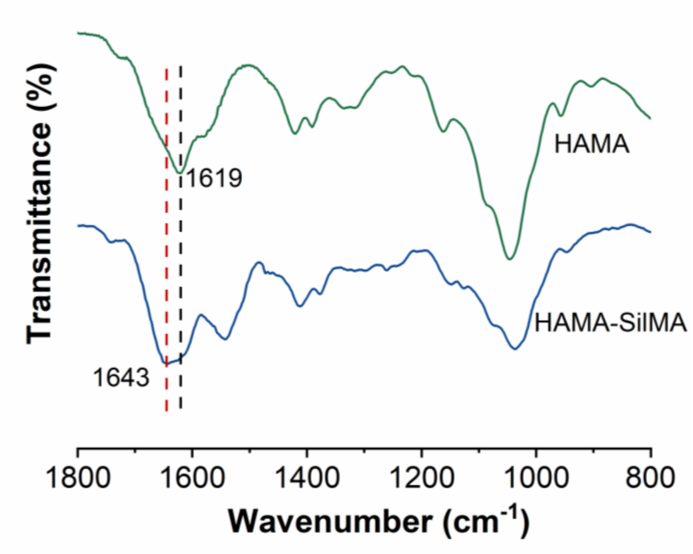

Supplementary Figure 5. **FTIR spectra of HAMA and HAMA-SilMA coating.** The FTIR spectroscopy showed a significant shift in the characteristic peaks of HAMA after the addition of SilMA, indicating the HAMA-SilMA composite formation.

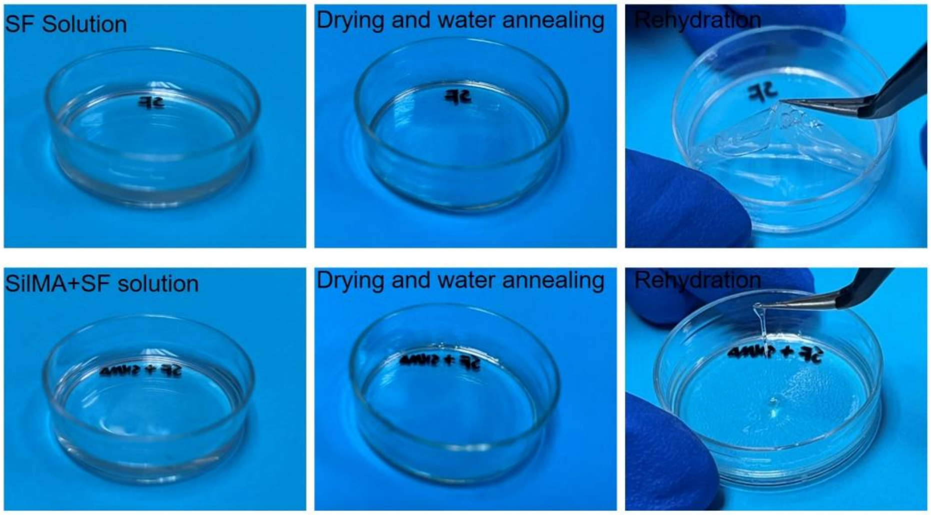

Supplementary Figure 6. **The SF and SFMA film after 2 min water annealing treatment.** The SF with higher MW and crystallinity had excellent structural and mechanical integrity, but its hydration ability was poor.

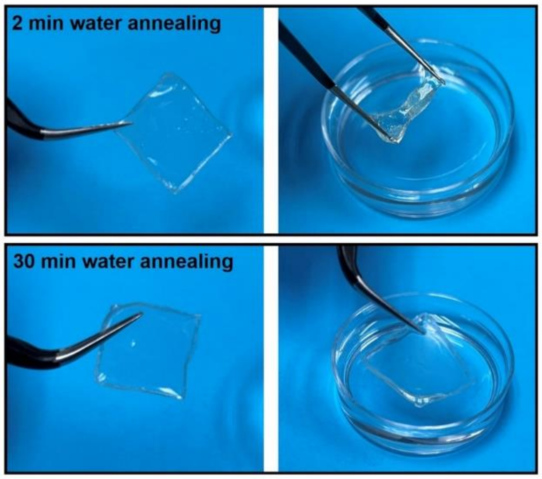

Supplementary Figure 7. The SFMA film was treated by water vapor annealing with different time. The SFMA film with longer treatment time was unable to hydrate.

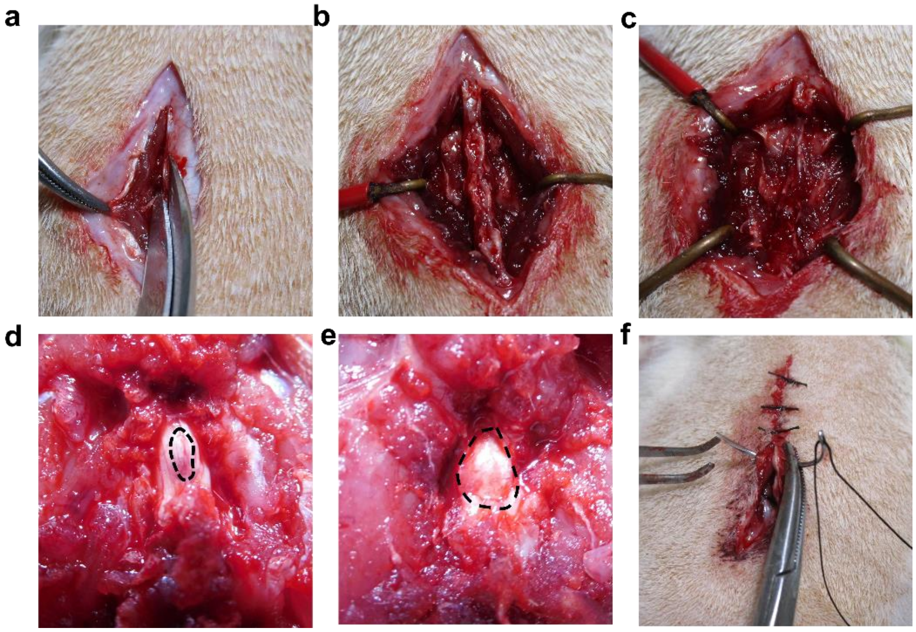

Supplementary Figure 8. The detail of surgical procedure. **a** A midline skin incision was made. **b** The spinous process was exposed. **c** The spinous process was cut. **d** The laminectomy was performed and the excision of dura mater was made. **e** Dural defect area covered with a Janus SIS membrane. **f** The muscle and skin were sutured. The black dashed line in (**d, e**) indicated the dural defect area and the implanted material, respectively.

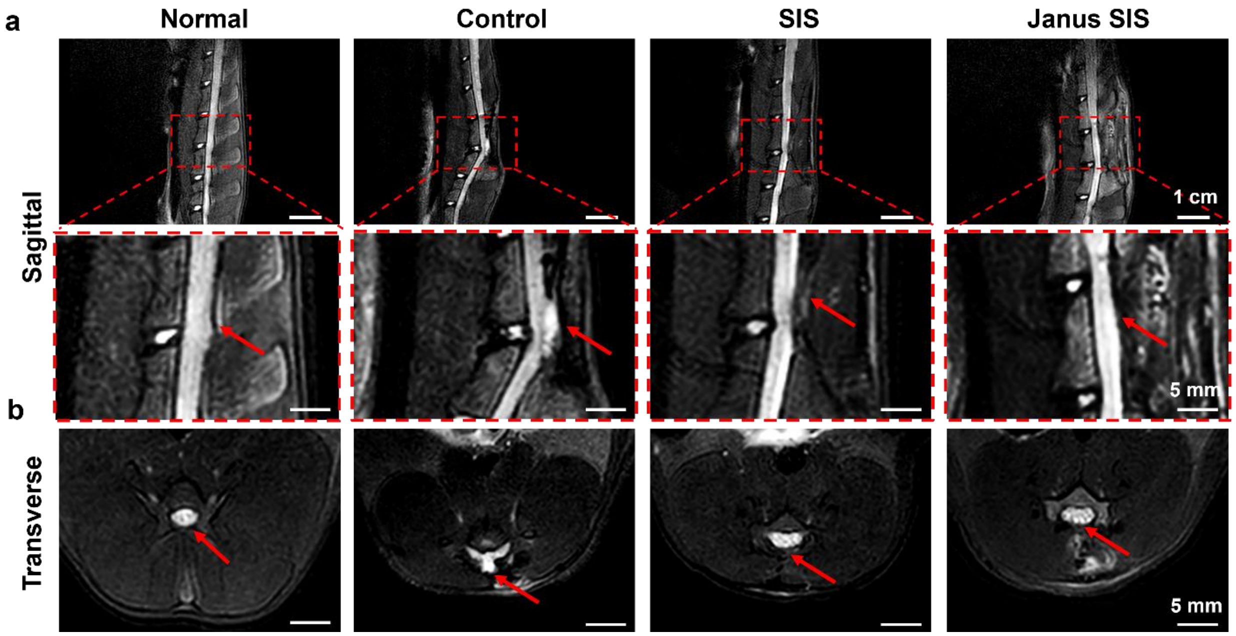

Supplementary Figure 9. The MRI images after 8 weeks surgery. **a, b** MRI images of the spine section 8 weeks after surgery with sagittal and transverse section. (Red arrow refers to the adhesion of the scar tissue to the spinal cord and the infiltration in the spinal canal.)
