# Supplementary material for: Janus decellularized membrane with anisotropic cell guidance and anti-adhesion silk-based coatings for spinal dural repair
Source: Nat Commun. 2025 Feb 15;16:1674. doi: 10.1038/s41467-025-56872-0 (PMC11829971; doi:10.1038/s41467-025-56872-0)
Supplement: Supplementary file 2 — Description of Additional Supplementary Files [file 41467_2025_56872_MOESM2_ESM.pdf]

Description of Additional Supplementary Files

File name: Supplementary Movie 1

Description: Videos on implanting Janus SIS membrane in vivo for repairing spinal dural defects. The rats were anesthetized and the back was disinfected, a midline incision on the back was made. Then, the lamina between L4-L5 was partial removed to expose the spinal dura mater, subsequently, dural defect about 3 mm×8 mm in size was created. The Janus SIS was implanted into the defect site.
