# Supplementary material for: Janus decellularized membrane with anisotropic cell guidance and anti-adhesion silk-based coatings for spinal dural repair
Source: Nat Commun. 2025 Feb 15;16:1674. doi: 10.1038/s41467-025-56872-0 (PMC11829971; doi:10.1038/s41467-025-56872-0)
Supplement: Supplementary file 4 — Reporting Summary [file 41467_2025_56872_MOESM4_ESM.pdf]

nature portfolio

Corresponding author(s): Xiaoming Li

Last updated by author(s): Feb 4, 2025

Reporting Summary

Nature Portfolio wishes to improve the reproducibility of the work that we publish. This form provides structure for consistency and transparency in reporting. For further information on Nature Portfolio policies, see our Editorial Policies and the Editorial Policy Checklist.

Statistics

For all statistical analyses, confirm that the following items are present in the figure legend, table legend, main text, or Methods section.

n/a	Confirmed	
☐	☒	The exact sample size (n) for each experimental group/condition, given as a discrete number and unit of measurement
☐	☒	A statement on whether measurements were taken from distinct samples or whether the same sample was measured repeatedly
☐	☒	The statistical test(s) used AND whether they are one- or two-sided<br>*Only common tests should be described solely by name; describe more complex techniques in the Methods section.*
☒	☐	A description of all covariates tested
☒	☐	A description of any assumptions or corrections, such as tests of normality and adjustment for multiple comparisons
☐	☒	A full description of the statistical parameters including central tendency (e.g. means) or other basic estimates (e.g. regression coefficient) AND variation (e.g. standard deviation) or associated estimates of uncertainty (e.g. confidence intervals)
☐	☒	For null hypothesis testing, the test statistic (e.g. F, t, r) with confidence intervals, effect sizes, degrees of freedom and P value noted<br>*Give P values as exact values whenever suitable.*
☒	☐	For Bayesian analysis, information on the choice of priors and Markov chain Monte Carlo settings
☒	☐	For hierarchical and complex designs, identification of the appropriate level for tests and full reporting of outcomes
☒	☐	Estimates of effect sizes (e.g. Cohen's d, Pearson's r), indicating how they were calculated

Our web collection on statistics for biologists contains articles on many of the points above.

Software and code

Policy information about availability of computer code

Data collection	No software was used
Data analysis	Origin 2018, peak fit 4.0 , IBM SPSS statistics 20.0, ImageJ and Image-Pro Plus 6.0.

For manuscripts utilizing custom algorithms or software that are central to the research but not yet described in published literature, software must be made available to editors and reviewers. We strongly encourage code deposition in a community repository (e.g. GitHub). See the Nature Portfolio guidelines for submitting code & software for further information.

Data

Policy information about availability of data

All manuscripts must include a data availability statement. This statement should provide the following information, where applicable:

- Accession codes, unique identifiers, or web links for publicly available datasets
- A description of any restrictions on data availability
- For clinical datasets or third party data, please ensure that the statement adheres to our policy

The authors declare that all relevant data supporting the findings of this study are available within this article and its supplementary information. All data are available from the corresponding authors upon request. Source data are provided as a Source Data file.

Research involving human participants, their data, or biological material

Policy information about studies with human participants or human data. See also policy information about sex, gender (identity/presentation), and sexual orientation and race, ethnicity and racism.

Reporting on sex and gender	We don't have related issues since this module is not applicable to my study.
Reporting on race, ethnicity, or other socially relevant groupings	We don't have related issues since this module is not applicable to my study.
Population characteristics	We don't have related issues since this module is not applicable to my study.
Recruitment	We don't have related issues since this module is not applicable to my study.
Ethics oversight	We don't have related issues since this module is not applicable to my study.

Note that full information on the approval of the study protocol must also be provided in the manuscript.

Field-specific reporting

Please select the one below that is the best fit for your research. If you are not sure, read the appropriate sections before making your selection.

☒ Life sciences ☐ Behavioural & social sciences ☐ Ecological, evolutionary & environmental sciences

For a reference copy of the document with all sections, see nature.com/documents/nr-reporting-summary-flat.pdf

Life sciences study design

All studies must disclose on these points even when the disclosure is negative.

Sample size	The sample size of each group for scanning electron microscopy (SEM)/confocal laser scanning microscopy CLSM/water contact angle test/ X-ray diffraction/Fourier-transform infrared spectroscopy/anti-swelling test /lap shear test/protein adsorption test/CCK-8 assay were three independent samples.The sample size was chosen considering the repeatability of experimental results and meet the requirements of scientific standards.<br>The sample size of each group for rheological test/tensile test were four independent samples considering the reproducibility of the experimental results and enhancing the reliability of the experimental results.<br>Three 6-8 weeks male BALB/c mice were used for in vivo degradation experiment, the f-HAMA-SilMA coating SIS group and f-HAMA coating SIS group were three independent replicates.<br>Six 6-8 weeks male Sprague-Dawley (SD) rats were used for the evaluation of histocompatibility, the SIS membranes with SFMA microgroove coating and HAMA SilMA coating groups consist of three independent replicates of each time points.<br>Three 6-8 weeks male SD rats were used for the observation of host cells arrangement, the SIS membranes with SFMA microgroove coating consist of three independent replicates.<br>Sixteen 6-8 weeks male SD rats were used for dura mater defect model:normal control (without defect modeling, four rats) , control group (defect modeling without membrane implantation, four rats) , SIS group (defect modeling with SIS implantation, four rats), and Janus SIS group (defect modeling with Janus SIS implantation, four rats).<br>These animal studies set three or four independent samples to support the findings of experimental results and meet the requirements of scientific standards.
Data exclusions	Data were not excluded from analysis.
Replication	All the quantitative data in this study has been repeated for as least 3 times independently. The n numbers are presented in each figure legend. All attempts at replication were shown with scatter in related figures.
Randomization	The samples in this experiment were randomly assigned.
Blinding	The data used for statistics in the article were measured in the same environment and there is no blinding issue.

Reporting for specific materials, systems and methods

We require information from authors about some types of materials, experimental systems and methods used in many studies. Here, indicate whether each material, system or method listed is relevant to your study. If you are not sure if a list item applies to your research, read the appropriate section before selecting a response.

Materials & experimental systems

n/a	Involved in the study	
	x	Antibodies
	x	Eukaryotic cell lines
x		Palaeontology and archaeology
	x	Animals and other organisms
x		Clinical data
x		Dual use research of concern
x		Plants

Methods

n/a	Involved in the study	
x		ChIP-seq
x		Flow cytometry
x		MRI-based neuroimaging

Antibodies

Antibodies used	collagen-1 Antibody; Boster; BA0325; rabbit polyclonal; WB ; IHC ; IF; IF/ICC;<br>CD68 Antibody; abcam; ab31630; mouse monoclonal ; Flow Cyt;<br>CCR7 Antibody; abcam; ab32527 ; rabbit monoclonal; WB; ICC/IF; IP; ELISA; IHC-P;<br>Anti-liver Arginase Antibody; abcam; ab91279; rabbit polyclonal; WB ; IP<br>Anti-alpha smooth muscle Actin Antibody; abcam; ab32575; rabbit monoclonal ; ELISA; Flow Cyt (Intra); IHC-Fr;WB; IHC-P; ICC/IF
Validation	collagen-1 Antibody:<br>https://www.boster.com/index/products/productsDetail?goods_sn=BA0325<br>CD68 Antibody:<br>https://www.abcam.cn/products/primary-antibodies/cd68-antibody-ed1-ab31630.html?productWallTab=Abreviews<br>CCR7 Antibody:<br>https://www.abcam.cn/products/primary-antibodies/ccr7-antibody-y59-ab32527.html<br>Anti-liver Arginase Antibody:<br>https://www.abcam.cn/products/primary-antibodies/liver-arginase-antibody-ab91279.html<br>Anti-alpha smooth muscle Actin Antibody:<br>https://www.abcam.cn/products/primary-antibodies/alpha-smooth-muscle-actin-acetyl-e3--actg2-acetyl-e3-antibody-e184-ab32575.html

Eukaryotic cell lines

Policy information about cell lines and Sex and Gender in Research

Cell line source(s)	Mouse embryonic fibroblasts (NIH-3T3, GNM 6, Chinese Academy of Sciences Cell Bank) were utilised for evaluating cell morphology, proliferation and collagen expression;
Authentication	Commercially purchased cell lines were not authenticated by study participants.
Mycoplasma contamination	All cell lines were tested negative for mycoplasma contamination.
Commonly misidentified lines (See ICLAC register)	No commonly misidentified cell lines were used in the study.

Animals and other research organisms

Policy information about studies involving animals; ARRIVE guidelines recommended for reporting animal research, and Sex and Gender in Research

Laboratory animals	6-8 weeks male BALB/c mice were used for in vivo degradation experiment; 6-8 weeks male Sprague-Dawley (SD) rats were used for subcutaneous implantation and dura mater defect model.
Wild animals	No wild animals were used in the study.
Reporting on sex	The male BALB/c mice and Sprague-Dawley (SD) rats were used for in animal experiments.<br>There are several reasons why male mice/rats are selected as subjects in scientific experiments:<br>1. Physiological differences: Female mammals have oestrous cycles that eventually turn into menstrual periods if they are not pregnant, potentially adding a tricky variable to scientific trials and data analysis. However, a considerable number of studies have also found that female mice do not respond differently to drugs at different times in the physiological cycle.<br>2. Ease of control: Male rats can be more easily controlled and standardized during the experiment because they do not have physiological changes such as the menstrual cycle.<br>3. Avoid confusion: The use of male animals can avoid the influence of physiological state changes such as estrus or pregnancy on the

	experimental results.
Field-collected samples	No field collected samples were used in the study.
Ethics oversight	All animal procedures were approved by the Biomedical Ethics Committee of Beihang University (Number: BM20200180)

Note that full information on the approval of the study protocol must also be provided in the manuscript.
